# The virtues and vices of protein citrullination

**DOI:** 10.1098/rsos.220125

**Published:** 2022-06-08

**Authors:** Maria A. Christophorou

**Affiliations:** The Babraham Institute, Cambridge CB22 3AT, UK

**Keywords:** protein, citrullination, peptidylarginine deiminase, disease

## Abstract

The post-translational modification of proteins expands the regulatory scope of the proteome far beyond what is achievable through genome regulation. The field of protein citrullination has seen significant progress in the last two decades. The small family of peptidylarginine deiminase (PADI or PAD) enzymes, which catalyse citrullination, have been implicated in virtually all facets of molecular and cell biology, from gene transcription and epigenetics to cell signalling and metabolism. We have learned about their association with a remarkable array of disease states and we are beginning to understand how they mediate normal physiological functions. However, while the biochemistry of PADI activation has been worked out in exquisite detail *in vitro*, we still lack a clear mechanistic understanding of the processes that regulate PADIs within cells, under physiological and pathophysiological conditions. This review summarizes and discusses the current knowledge, highlights some of the unanswered questions of immediate importance and gives a perspective on the outlook of the citrullination field.

## 1. Introduction

Protein expression is subject to several, super-imposing layers of regulation. Transcriptional, post-transcriptional and translational control mechanisms determine whether a protein is expressed within a certain cell, at a certain time. Once a protein is translated, it can be chemically modified through enzymatic and non-enzymatic reactions. These post-translational modifications (PTMs) can impact the structure, stability, sub-cellular localization and activity of a protein and modulate its binding affinity to other proteins, metabolites and nucleic acids. Protein modifications can be spatially and temporally controlled, allowing cells to respond to environmental changes such as stress signals, developmental cues, changes in nutrient or oxygen availability and oncogenic insults. PTMs, therefore, expand the functional proteome far beyond the complexity of the genome, and add an enormous degree of sophistication to biological systems, making them responsive and adaptable. Over 200 types of PTMs have been described to date [1].

Citrullination, or peptidylarginine deimination, is the post-translational conversion of an arginine residue to citrulline and involves the hydrolysis of the arginine and concomitant release of ammonia. Citrullination converts the guanidinium group of arginine to a ureido group, resulting in the loss of positive charge and two potential hydrogen bond donors [2]. Depending on the location of the modification within the protein, this may have profound consequences for protein function by altering local electrostatic interactions and hydrogen bonding ability. Indeed, like other PTMs, citrullination has been shown to impact several aspects of protein biology, such as structure, stability, localization, protein and nucleic acid binding and catalytic activity, as well as affect the subsequent deposition of other PTMs. As citrulline is a non-coded amino acid, its presence within a protein can only result through modification, implying a change in the cell's state or environment and the initiation of a relevant response.

Citrullination is catalysed by a small family of enzymes, the peptidylarginine deiminases (PADIs or PADs). The five PADI family members, PADI1, 2, 3, 4 and 6, are structurally similar and likely to operate via common regulatory mechanisms, but they show varying tissue distributions and sub-cellular localizations, suggesting that they have specific and non-overlapping organismal roles [3,4]. A large number of citrullinated proteins have been identified in different biological and disease systems [5–10] and this is likely to be a reflection of the wide regulatory capacity of citrullination. It is noteworthy however that, unlike kinases, ubiquitinases, methyltransferases or acetyltransferases, PADIs comprise a very small family of enzymes, with only four proteins having catalytic activity. This, coupled with their remarkably strong association with the development of pathology, makes it worth considering how PADIs are regulated and which molecular and cellular functions they modulate in health and disease.

Although the presence of citrulline within proteins has been known for nearly 50 years [11], having been suggested as early as the 1930s [12], and the first PADI was isolated 30 years ago [13], citrullination has been a rather obscure PTM for a long time. Historically, PADIs have been best known for their role in disease development. Research over many years has established strong and, in some cases, causal associations between aberrantly high levels of citrullination and the development of autoimmunity, neurodegeneration and cancer [14–16]. Conversely, lack of PADIs is associated with defects in embryo development, neurodevelopment and infertility [10,17,18]. The involvement of deregulated citrullination in disease has necessitated the development of biochemical, proteomic and computational methods for its detection [5,8,19–22], as well as pharmacological approaches for its inhibition [23,24], and important progress has been achieved in these areas.

A lot remains to be understood about the organismal functions of PADIs, the molecular and cellular mechanisms that underlie their physiological roles and how these relate to the deregulation of the modification in disease. Central to this is understanding the precise mechanisms of PADI activation within cells in different physiological and pathophysiological contexts. This review will summarize our current understanding, discuss some of the immediate open questions and provide a perspective on the future outlook of the field.

## 2. Distribution of citrullination across the tree of life and evolution in animals—*an evolutionary accident?*

The distribution of PADIs across the tree of life is highly unusual and the PADI sequence has been subject to extensive losses, modifications and duplications across evolution [25]. PADIs are present in some bacteria and fungi and, although their functions in these organisms are completely unknown, they have been shown to be catalytically active [25–27]. Paradoxically, PADIs are absent from yeast, worms and flies, but are ubiquitous across vertebrates. Ray-finned fish have a single *PADI* gene, but duplications down the vertebrate lineage have resulted in five paralogues in mammals. At least two different PADI types can be found across life [25], the animal-type three-domain PADI and the fungal-type, two-domain PADI. The animal-type PADI emerged during cyanobacterial evolution, while the fungal-type PADI can be found in actinobacteria and other bacteria. This and other complementary pieces of evidence, including phylogeny and sequence evolution rate analyses, indicate that the ancestral cyanobacterial gene was introduced into animals by horizontal gene transfer [25]. The cyanobacterial PADI is catalytically active and can citrullinate mammalian proteins including histones, which are absent from bacteria, suggesting that the horizontal transfer event introduced citrullination as a new catalytic activity in animals.

Two other types of enzymes can catalyse citrullination: pPAD, an extended agmatine deiminase found in the human pathogen *Porphyromonas gingivalis* [28] and gADI, an extended free L-arginine deiminase found in the human parasite *Giardia Lamblia* [29]. These have evolved independently from PADIs and are found in bacteria and some eukaryotic organisms [25]. Furthermore, a recent study identified a protein with peptidylarginine deiminase activity in the plant *Arabidopsis thaliana* [30]*.* Protein At5g08170 was identified after searching the *Arabidopsis thaliana* genome for a protein motif generated by aligning the catalytic core sequences of bacterial deiminases, after the authors identified citrullinated proteins within the *Arabidopsis* proteome. It is unclear whether At5g08170 is a PADI homologue, since a comprehensive search for PADI orthologues across the tree of life identified no PADI homologues in plants [25]. Regardless of this, however, At5g08170 was shown to act in a calcium-dependent manner, similarly to PADIs, to citrullinate proteins with nucleic acid functions, upon cold stress. The authors suggest that citrullination in plants is responsive to stress and may be associated with cell reprogramming, although more evidence is required to support this suggestion.

It is therefore intriguing that citrullination, as a catalytic function, has emerged in a variety of ways across evolution (via PADIs, pPAD, gADI and At5g08170). Further research into the function of the enzymes described above, in their corresponding host organisms, will ascertain whether they operate via similar underlying molecular principles and have similar cellular functions across different species (e.g. modulation of histone function or cellular reprogramming). This is a fascinating area of research and an exciting next frontier for the citrullination field.

## 3. Structure, sub-cellular localization, tissue distribution and substrate specificities of PADIs*—a PADI for nearly every cell*

The PADI family enzymes exhibit high protein sequence homology, both between the five paralogues (greater than 50%), as well as between orthologues from different mammalian species [4,25]. Human PADIs consist of three structural domains, the N-terminal (PAD_N, Pfam annotation: PF08526), middle (PAD_M, Pfam annotation: PF08527) and catalytic C-terminal domain (PAD_C, Pfam annotation: PF03068). PADI2, 3 and 4 are active as head-to-tail homodimers, where the N-terminal domain of one monomer is in contact with the C-terminal domain of the other [3,4,31,32]. PADI1-4 are calcium-dependent and have highly conserved calcium binding and catalytic residues, while PADI6 lacks some of the calcium-binding residues and the catalytic cysteine [3,4]. PADI6 is therefore considered to be enzymatically dead and, to the author's knowledge, no PADI6 protein substrates have been identified to date.

PADIs differ in their sub-cellular localization and tissue distribution. PADI1, 3 and 6 localize in the cytoplasm, PADI2 can shuttle between the cytoplasm and the nucleus and PADI4, the only PADI that possesses a *bona fide* nuclear localization signal (NLS), is found predominantly in the nucleus [33,34]. Intriguingly, PADI4 has also been shown to be exposed on the cell surface of resting human neutrophils and PADI2 to be released to the extracellular space by the same cells [35]. Although it remains to be determined whether these findings translate across different cell types, they open new possibilities regarding the regulatory scope of PADI2 and PADI4.

Under physiological conditions, PADIs show highly variable tissue-specific distribution. PADI1 is expressed in the skin and the endometrium [36,37], while expression of the protein in the esophagus, testis, kidney and cervix is also suggested in the Human Protein Atlas [38]. PADI2 expression is most widespread and is found in the brain, uterus, spleen, pancreas, skeletal muscle and secretory glands [39,40], while Human Protein Atlas additionally suggests expression in the human digestive and gastrointestinal tract, kidney, bladder, testis and bone marrow [38]. In fact, Human Protein Atlas suggests some level of PADI2 mRNA expression in virtually all tissues, making it possible that all tissues have the potential for PADI2 expression under certain conditions, or within certain cell sub-populations. The fact that PADI2, which is the ancestral vertebrate *PADI* paralogue [25], is also the most widely expressed, may suggest that the remaining PADIs, which arose by duplication, were selected during evolution to carry out specific functions. Support for this idea comes from the finding that, while PADI2 expression in the pituitary is not indicated by the Human Protein Atlas, it has been shown to increase in this tissue during the estrous cycle [41]. PADI3 expression is restricted mainly to the skin and hair follicles [42–44]. PADI4 (also known as PAD V) is only strongly expressed in the bone marrow, spleen and blood, with highest expression in monocytes and granulocytes and some expression in haematopoietic progenitor cells [33,45,46], while emerging evidence demonstrates that it is also expressed in the uterus, sperm, oocytes and mammalian embryos [10,47]. Like PADI2, however, data from Human Protein Altas suggests that PADI4 mRNA is detectable at low levels in cell sub-populations of most tissues [38], suggesting that its expression and, possibly activation, is possible upon certain stimuli. PADI6 (also known as ePAD), is expressed in oocytes and mammalian embryos [48]. While PADIs show a well-defined tissue distribution under physiological conditions, their tissue expression can be highly deregulated in disease [49].

Although PADIs share high structural homology, they have different substrate specificities *in vivo* [50,51]. The structural basis for substrate recognition specificities is not yet understood. The differing substrate preferences may be a consequence of their differing sub-cellular localizations and expression patterns, but may also be determined by whether conditions are permissive for their activation in certain cellular contexts, for example, the presence of cofactors or inhibitory interactions. We don't have sufficient information at present to deduce general principles about PADI target specificities, such as consensus amino acid sequence motifs, however our ever-increasing understanding and advancements in proteomic and analytical methodologies, along with better biochemical understanding of their regulation *in vivo*, is likely to yield these insights in the years to come.

## 4. Molecular and cellular functions regulated by citrullination—*a small family with a big reach*

We are gaining an ever-increasing understanding of the molecular and cellular processes impacted by citrullination. This knowledge has come both from large-scale proteomic studies, which have aimed to deduce general principles through identifying the ‘citrullinome’ [5,6,8], as well as targeted studies that have examined the role of PADIs and citrullination of specific substrates.

In a recent review, Genander *et al*. analysed published human citrullinomes of cells under resting and inflammatory conditions and identified the gene ontology (GO) categories represented most highly [52]. They demonstrate that, in resting cells, citrullinomes are enriched for proteins involved in RNA metabolic processes and gene expression, while under inflammatory conditions they are overwhelmingly enriched for GO categories relating to immune responses [52]. This may be a manifestation of the magnitude of PADI activation achieved in the two different scenarios, while it is also worth considering the conditions under which the citrullinome was measured. For example, in studies that probed the citrullinome under conditions of aberrant activation (for example in autoimmune disease or upon activation with broad stimuli, such as calcium ionophores), many proteins that become citrullinated are not PADI targets under physiological conditions. In this respect, we note that the GO categories found to be enriched within the citrullinomes of resting cells generally comprise highly abundant proteins, such as cytoskeletal and ribonuclear proteins [52]. Advances in mass spectrometric methods and associated computational analysis tools will undoubtedly lead to the identification of low abundance substrates, or citrullination events that may be present in sub-stoichiometric concentrations but nonetheless be functionally relevant, in the not too distant future. A better understanding of the mechanisms that govern PADI activation *in vivo*, beyond inflammation, will also allow researchers to tailor experiments so they reveal classes of PADI substrates that are relevant in new contexts.

For the purposes of this review, the molecular and cellular processes impacted by PADIs, as have been studied through specific protein substrates, are discussed.

### 4.1. Gene regulation

PADIs modulate gene expression in a number of ways: (i) via histone citrullination; (ii) via citrullination of transcription factors and epigenetic regulators; and (iii) via citrullination of signalling mediators that impact on gene expression. In addition, citrullination can modulate the likelihood and functional consequences of other PTMs on neighbouring amino acids.

#### 4.1.1. Histone citrullination

Core histones represent one of the prototypical PADI substrate classes [53] and citrullination of the N-terminal tails of histones is intimately linked to transcriptional regulation. PADI4-mediated citrullination of histone H3 at residues Arg2, Arg8 and Arg17 (H3R2/8/17) and histone H4 at Arg3 (H4R3) were initially shown to repress transcription through the *pS2* promoter by antagonizing CARM-1-mediated arginine methylation [54,55]. A subsequent study, which probed PADI4 localization and activity more globally, found that it is primarily associated with gene activation, as PADI4 localizes near the transcriptional start sites of active genes and its binding correlates with the binding of activating transcription factors, while it anti-correlates with repressive chromatin modifications [16]. In addition, PADI2-mediated citrullination of histone H3 Arg26 (H3R26) has also been shown to promote the transcriptional activation of estrogen receptor *α* (ER*α*) target genes in breast cancer [56,57] and of interleukin 6 (IL-6) in multiple myeloma [58]. Further support for the role of histone citrullination as a gene regulation mechanism comes from work in leukaemia cells, which demonstrated that PADI4 acts as a co-activator of translocated in leukaemia 1 (Tal1) through citrullination of H3R2 [59]. Beyond mediating gene regulation, histone citrullination was also recently shown to regulate non-coding RNA expression in pituitary cancers [60].

Although the above studies demonstrated disease-associated, aberrant PADI-mediated transcriptional regulation, this has also been demonstrated in physiological contexts. A study of normal canine mammary epithelial tissue suggested that PADI2-mediated H3 citrullination may regulate lactation-related genes during diestrus [61]. Additionally, PADI2-mediated citrullination of H3R26 mediates the expression of genes involved in oligodendrocyte differentiation [18], while PADI4-mediated H3 citrullination regulates the expression of pluripotency genes in embryonic stem cells [10].

Histone modifications act as a platform for the binding of transcriptional regulators. It is, therefore, possible that citrullination ‘readers’ bind histone citrulline marks and direct transcriptional protein complexes to specific genomic sites. Support for this mechanism comes from a study that showed that citrullinated H3R26 (H3Cit26) is bound by the chromatin remodelling factor Smarcad1 in pluripotent stem cells [62]. On the other hand, citrullination can modulate the binding of transcription factors and other chromatin-associated proteins to nearby histone marks and has been shown to work in conjunction with lysine methylation and lysine acetylation. Citrullination of H3R8 reduces the binding of heterochromatin protein 1 (HP1) to sites carrying trimethylation of H3 lysine 9 (H3K9me3), a prototypical heterochromatic mark, resulting in de-repression of cytokine genes and human endogenous retroviruses [63]. In addition, histone H3 citrullination was shown to be coordinated with H3 lysine deacetylation, through association between PADI4 and histone deacetylase 1 (HDAC1) during repression of the *pS2* promoter in cancer cells [64] and in haematopoietic progenitor cells [65], suggesting that the crosstalk between citrullination and deacetylation may operate more broadly. It is likely that many more instances of citrullinated-histone-associated factors and interplay between citrullination and other PTMs will be uncovered in the future. Provided that PADI2 and PADI4 have clear regulatory roles in development and disease, systematic approaches for the identification of citrullination readers are warranted. In addition, it is exciting to consider how citrullination may play into the ‘histone code’ [66].

Citrullination of linker histones impacts chromatin structure. PADI4-mediated citrullination of linker H1 at Arg54 (H1R54), which is positioned within the H1 globular domain, results in reduced association with nucleosomal DNA and chromatin decondensation in mammalian embryonic stem cells and neutrophils [10,67], while similar results have been obtained with the avian H1 orthologue, histone H5 [68]. Citrullination of linker histones mediates both the local decondensation associated with a transcriptionally permissive chromatin state, and the global and dramatic chromatin decompaction associated with Neutrophil Extracellular Trap (NET) formation (discussed in detail below). The factors that determine the degree of linker histone citrullination and the subsequent degree of chromatin decondensation are unclear, however, it is likely that the nature or magnitude of PADI activation differs between the two scenarios. As discussed above, understanding the mechanistic details of PADI activation within the nucleus will not only allow us to understand this dichotomy but also potentially allow us to manipulate the process to achieve a desired degree of chromatin decondensation.

#### 4.1.2. Citrullination of transcriptional and epigenetic regulators

In addition to modifying histones, PADIs can regulate transcription via citrullination of transcription factors and epigenetic regulators and by acting as transcriptional cofactors. PADI4-mediated citrullination of Elk-1, a transcription factor and downstream target of MAPK-ERK signalling, facilitates its phosphorylation by ERK2, which in turn increases its association with the histone methyltransferase p300, ultimately leading to transcriptional activation of target genes [16]. An independent study reported that, in 293T cells, PADI4 can target p300 directly, resulting in its enhanced association with co-activator GRIP1 [69].

Additionally, citrullination can impact epigenetic state through modulation of the DNA methyltransferases (DNMTs). In cancer cells, PADI4-mediated citrullination of the de novo methyltransferase DNMT3a results in its stabilization and increased DNA methylation at certain promoters [70]. Citrullination of DNMT3b has also been reported in mouse embryonic stem cells [10], suggesting this may be a more widely applicable mechanism. Notably, citrullination of RNA polymerase II (RNApolII) has been shown to impact on gene transcription [71]. In breast cancer cells, PADI2 citrullinates the C-terminal domain (CTD) of RNApolII, which acts as a regulatory hub where PTMs mediate the binding of auxiliary factors, determining polymerase progression [72]. Citrullination of the RNApolII mediates its association with the positive transcription elongation factor b (P-TEFb) kinase complex and its recruitment to chromatin, regulating RNApolII processivity.

It is unclear whether the above mechanisms operate in normal cells and are co-opted by cancer cells upon positive selection of PADI upregulating mutations. An alternative scenario is that such mechanisms only operate within transformed cells, where deregulated PADIs have the capacity to acquire new protein targets (due to a breakdown in the regulation of their levels or activity) and ‘hijack’ additional molecular pathways. The above studies have generated sufficient knowledge and novel reagents, such as citrullination-specific antibodies, to render possible comparative studies between normal and cancer cells, allowing deeper understanding of how PADIs impact on cancer initiation and progression.

Beyond studies in cancer cells, a significant amount of our current knowledge on citrullination-mediated transcriptional regulation comes from studies of cytokine gene regulation in inflammatory cells. PADI4-mediated citrullination of the transcription factor E2F1, cooperates with its acetylation to enhance its binding to the BET family bromodomain reader BRD4 (bromodomain-containing protein 4) [73]. This facilitates binding of E2F1 specifically to cytokine genes within granulocytes during inflammation. Additionally, PADI4-mediated citrullination of the nuclear factor *κ*B (NF*κ*B) p65 promotes its nuclear localization and transcriptional activity by enhancing its interaction with importin *α*3 [74]. The PADI4-mediated and enhanced transactivation activity of NF*κ*B is specifically targeted to inflammatory cytokine genes interleukin 1*β* (IL1*β*) and tumor necrosis factor *α* (TNF*α*), promoting inflammation. Additionally, PADI2 was shown to directly citrullinate transcription factors GATA3 and ROR*γ*t and modulate their DNA binding activity, ultimately skewing T-cell differentiation outcomes [75].

#### 4.1.3. PADI recruitment to chromatin

Although the effects of histone citrullination on transcription and chromatin compaction are well documented and we are beginning to map the mechanisms underlying this type of regulation, the mechanisms that govern the association of PADIs with chromatin remain unknown. PADIs do not possess a *bona fide* DNA binding domain and we currently lack data that will allow us to ascertain whether they preferentially associate with specific DNA sequences. Binding to specific genomic sites may be mediated by the DNA sequence itself, by other histone modifications, or by association with other transcriptional regulators or protein complexes. An example of the latter comes from studies on the regulation of ERα-mediated transcription, where it was found that stimulation of breast cancer cells with 17β-estradiol leads to association of PADI2 with ER*α* and recruitment of PADI2 to ER*α* target promoters [56]. Notably, in this system PADI2-mediated histone citrullination is observed mere minutes after stimulation with 17β-estradiol and facilitates ER*α* binding to chromatin, suggesting that it is one of the initiating events in ER*α*-mediated transcriptional regulation [57]. It is therefore unclear at this point whether additional factors may mediate association of PADI2 with DNA, or whether this is mediated exclusively by ER*α*. The complexity of these scenarios, coupled with the fact that chromatin-associated PADIs may, in principle, be catalytically active or inactive under different conditions, necessitates detailed studies that will allow us to precisely map PADIs and their activation across the genome under different cellular stimuli. This will undoubtedly be facilitated by the generation of new and specific reagents, such as anti-PADI and citrullination-specific antibodies.

In addition to modifying chromatin and associated proteins, PADIs may modulate the transcriptional and epigenetic state of cells indirectly, through modulation of signalling pathways that affect DNA-based events. The emerging role of citrullination in signal transduction is discussed next.

### 4.2. Cell signalling

A number of studies have started to integrate citrullination within cell signalling cascades. PADIs are activated by inflammatory signalling and impact upon it. In addition to PADI-mediated transcriptional regulation of cytokine genes, which is described above, citrullination of cytokines has also been shown to biochemically regulate their function. PADI2 citrullinates chemokines CXCL10 and CXCL11 and reduces their chemoattracting capacity, as well as their ability to signal through the chemokine receptor CXCR3, through a mechanism that's independent of receptor binding impairment [76]. Similarly, citrullination of CXCL8/IL-8 results in reduced CXCR2-mediated signaling [77], while citrullination of CXCL12 at different sites differentially modulates its binding to receptors CXCR4 and CXCR7 [78]. Furthermore, TNF*α* citrullination leads to reduced production of chemokines CXCL8 and CXCL10 [79]. The above findings seem paradoxical when considered against the strong association between PADI activation, inflammation and inflammatory disorders, as well as the evidence for PADI-mediated transcriptional activation of cytokine genes [58,63,73,74].

PADIs have also been implicated in signal transduction pathways that regulate cell growth, invasiveness and the transition between the epithelial and mesenchymal states. PADI4 citrullinates glycogen synthase kinase-3*β* (GSK3*β*) and mediates its translocation to the nucleus, thereby dampening TGF*β* (transforming growth factor beta) signalling, while knock-down of PADI4 in this system promotes epithelial-to-mesenchymal transition and enhances the metastatic potential of cancer cells [80]. In addition, PADI2 was shown to inhibit Wnt signalling through citrullination of β-catenin, which enhances its degradation in a manner that's independent of its GSK3β-mediated regulation [81]. These two studies therefore describe anti-tumorigenic functions of PADI2 and PADI4, through dampening of cancer promoting signal transduction pathways. On the other hand, it was shown that PADI1 is over-expressed and may promote development of triple-negative breast cancer (TNBC) through direct citrullination of MEK1 kinase and modulation of its potential to phosphorylate ERK1/2 [82]. A study by the same laboratory showed that MEK1 is targeted by PADI2 in endometrial cancer and the citrullination event promotes MEK1-mediated phosphorylation of ERK1/2, facilitating tumour progression [83].

The implication of citrullination in classical signal transduction pathways is both fascinating and daunting and the fact that these pathways are highly pleiotropic may complicate, rather than clarify, our understanding of the regulation and roles of PADIs. As discussed above, future efforts in the field may build on these studies to assess whether PADIs impact on growth promoting signaling pathways under conditions of homeostasis.

### 4.3. Cell and tissue structure

PADIs impact on cell and tissue robustness through citrullination of structural proteins. PADI1- and PADI3-mediated citrullination of structural and intermediate filament-associated proteins such as keratins, trichohyalin and filaggrin provide mechanical strength and contribute to the maintenance of healthy skin and hair follicles, while their deregulation is associated with skin diseases such as psoriasis [84–88]. PADI2-mediated citrullination of the ECM protein Fibulin-5 protects it from proteolytic degradation and maintains elastogenic tissue function in the lungs [89]. Furthermore, citrullination of collagen type I is thought to mediate mesenchymal-to-epithelial transition [90], promoting cancer metastasis, while citrullination of fibronectin was also shown to promote cell invasion through altering integrin-mediated signalling [91]. Similarly, citrullination of collagen type II modulates its binding to integrins and decreases cell adhesion [92]. Finally, PADI6 localizes to the intermediate filament structures of oocytes and mediates cytoskeletal integrity [48,93].

Another type of structural protein whose function is regulated by citrullination is Myelin Basic Protein (MBP), a major constituent of the nerve myelin sheath and essential in the transmission of action potentials and motor function [94]. A significant body of work has determined that the correct level of citrullination is essential for proper MBP function. Aberrantly high levels of MBP citrullination are associated with the development of multiple sclerosis (MS) and have been shown to lead to destabilization of the myelin sheath [15,95].

The above studies demonstrate that precise regulation of PADI activity is essential for the maintenance of healthy tissue function. PADI inhibition has been suggested as a therapeutic strategy for autoimmune and neurodegenerative disorders, as well as cancer, due to the fact that these disease states are typically associated with aberrantly high levels of citrullination. However, caution is warranted to ensure therapeutic interventions that perturb the activity of PADIs are developed on solid understanding of their roles in tissue physiology.

### 4.4. Emerging functions

Recent work has implicated citrullination in a number of new biological processes. These are discussed briefly below.

#### 4.4.1. Cell metabolism

A study published last year showed that PADI1- and PADI3-mediated citrullination of the glycolytic enzyme pyruvate kinase M2 (PKM2) alters its binding to amino acids and promotes glycolysis in cancer cells [96]. A more recent study showed that citrullination of glukokinase leads to reduction of its catalytic activity and results in reduced insulin secretion in response to glucose stimulation [97]. These are, to the author's knowledge, the first reports that mechanistically link protein citrullination to the regulation of metabolic pathways. However, the first study identified several citrullinated proteins within the glycolytic pathway, opening up the possibility that PADIs can impinge on cellular metabolism more broadly.

#### 4.4.2. RNA biology

Another recently reported function of citrullination is in RNA metabolic processes. As mentioned above, proteins within the GO categories relating to RNA biology are highly represented within citrullinome datasets [52], but a functional role for PADIs in such processes has not been studied widely. Citrullination of the splicing factor PSF was shown to regulate its binding to mRNA [98]. In this respect, it is notable that a significant subset of citrullinated proteins identified in one study contain the RG/RGG RNA binding motif [9]. This particular study showed that PADI4-mediated citrullination inhibits arginine methylation of those proteins and prevents their aggregation. However, these findings open the possibility that citrullination of RNA binding motifs may be a more general mechanism of regulating the function of protein-RNA binding.

The fact that conversion of arginine to citrulline removes a positive change, which would be predicted to modulate interactions with charged molecules such as nucleic acids or certain charged metabolites, suggests that wider roles of citrullination in the regulation of metabolism or RNA-mediated processes are plausible. Formal experimentation will determine whether this is indeed the case and whether such mechanisms operate under physiological conditions or only become relevant under conditions of PADI deregulation.

#### 4.4.3. Autophagy

Functional links are also emerging between citrullination and autophagy. Induction of autophagy has been shown to lead to activation of PADI4 and generation of citrullinated neo-epitopes that are associated with autoimmunity [99], while processing of proteins via autophagic vesicles was shown to lead to the generation and presentation of citrullinated peptides specifically, by antigen presenting cells, synoviocytes and fibroblasts [99,100]. It is not understood how activation of autophagy leads to citrullination. PADI activation within autophagic vesicles may be a result of increased local calcium availability, however further investigation is required to ascertain whether specific autophagy-associated signalling can modulate PADI activity. On the other hand, PADIs have been implicated in the regulation of autophagy [101–103], suggesting that PADI activation may happen independently of autophagy signalling. Understanding the mechanistic details of the interplay between autophagy and citrullination may provide new ways of modulating autophagy in cancer therapy and new avenues of alleviating tissue destruction in autoimmunity.

## 5. Citrullination in health and disease*—the virtue and the vice*

**Figure 1.**
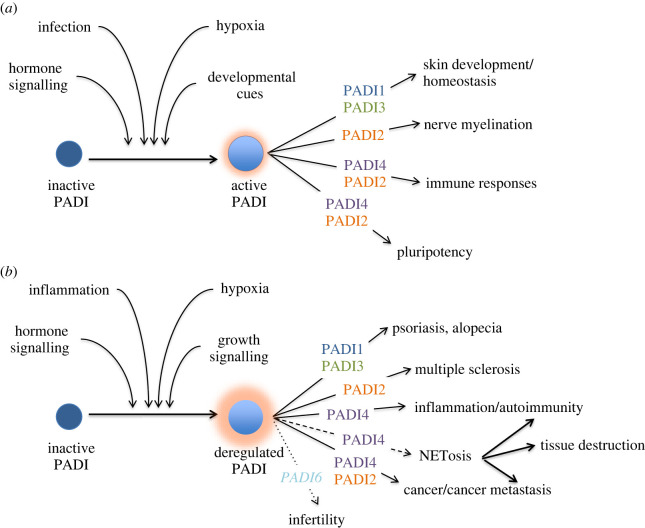
Organismal functions of citrullination in physiology and disease. (*a*) Under physiological conditions, PADIs are activated in response to stimuli such as infection, hormone stimulation, developmental signals and, possibly, hypoxia. Depending on their tissue expression, PADIs can regulate skin homeostasis, nerve myelination, immune responses and embryo development. PADI6, which is considered to be catalytically inactive, is not included in this schematic. (*b*) When one of the activating signals or the level of PADI activity are deregulated, aberrant levels of citrullination can underlie the development of skin disorders, multiple sclerosis, cancer progression, metastasis and autoimmunity. Deregulation of NETosis can exacerbate autoimmunity, cancer metastasis and tissue destruction associated with a number of disease states. Lack of PADI6 leads to compromised female fertility.

The last decade has seen significant progress in our understanding of the physiological functions of PADIs in health and disease. Depending on their tissue distribution, different PADIs have been shown to modulate the innate immune response, skin homeostasis, nerve myelination, stem cell biology, fertility and reproductive functions (figure 1). Conversely, deregulation of PADI levels or PADI activating pathways may lead to a breakdown in the tight regulation of PADI enzymatic activity and result in the non-physiological citrullination of additional proteins and disruption of their normal functions. This is illustrated in the fact that aberrant PADI activity is associated with an increasingly broad array of diseases, from autoimmunity and neurodegeneration to atherosclerosis and cancer (figure 1).

### 5.1. Neurobiology and neurodegeneration

Citrullination has been studied both in the context of central nervous system (CNS) physiology and neurodegeneration [94]. PADI2 is the most highly expressed PADI in the brain and CNS [18,104]. Its function as a transcriptional regulator is required for oligodendrocyte differentiation, while a tightly regulated level of citrullination of MBP maintains myelin sheath stability, as demonstrated by the fact that PADI2-KO mice display motor dysfunction [18]. On the other hand, aberrantly high levels of MBP citrullination also destabilize myelin and are thought to underlie the development of MS, as described above. This is supported by *in vivo* studies, which demonstrated that mice that over-express PADI2 in oligodendrocytes exhibit myelin loss [15]. In addition, PADI-mediated inflammation in the brain and PADI4-mediated transcriptional deregulation in oligodendrocytes have also been suggested as mechanisms underlying the development of MS [105]. In this respect, PADI inhibition has been suggested as a therapeutic strategy against MS [106]. Aberrantly high levels of citrullination have also been associated with neurodegenerative disorders such as Alzheimer's and prion diseases [107,108], however, a clear mechanistic understanding of how PADI deregulation may mediate disease progression in these cases still needs to be developed.

### 5.2. Skin homeostasis and skin diseases

A detailed review of the roles of PADIs in skin homeostasis and discussion of PADI-mediated mechanisms that may underlie skin diseases has recently been published elsewhere [109]. Briefly, PADI1 and PADI3 are expressed in the epidermis and hair follicles, where they mediate skin cell differentiation and maintain epidermal barrier function through citrullination of skin structural proteins [86]. It is not yet clear whether histone citrullination-mediated transcriptional regulation has a role in skin differentiation and homeostasis [52]. Genetic Polymorphisms of PADI3 are associated with skin disorders such as uncombable hair syndrome and certain forms of alopecia [110,111], while reduced levels of keratin K1 citrullination are thought to compromise skin structure and are associated with psoriasis and atopic dermatitis [87].

### 5.3. Reproductive biology, fertility and embryo development

PADIs have a regulatory role in the female reproductive system [112]. As described above, PADI6 is specific to the oocyte and is essential for female fertility through maintaining the stability of the oocyte cytoskeleton [48,93]. PADI2 and PADI4 have been shown to regulate the expression of insulin-like growth factor-binding protein 1 (IGFBP1) in response to progesterone, in the uterine cells of pregnant sheep [113,114]. As IGRBP1 is important for embryo implantation, it is suggested that PADI activity is important for the establishment of pregnancy. Further indication that PADIs may regulate female fertility comes from the finding that PADI2 expression in the mouse pituitary shows cyclic expression according to the estrous cycle [41].

Citrullination also plays a role in embryo development. PADI4 and citrullinated H3 are detectable in the mouse and pig pre-implantation embryo from the two-cell-stage onwards and PADI inhibition leads to a reduction in the number of pluripotent cells of the Inner Cell Mass (ICM), while PADI4 also regulates the expression of pluripotency genes in embryonic stem cells [10,115–117]. PADI4-KO mice are viable, but are born with skewed Mendelian ratios [118], suggesting that loss of PADI4 compromises embryonic development. It is possible that the loss of PADI4 is compensated by one of the other PADI members, for example PADI2 which can also mediate some of the same transcriptional mechanisms [18], however mouse models that are null for more than one PADI are required to ascertain this.

### 5.4. Immunity, inflammation and autoimmunity

**Figure 2.**
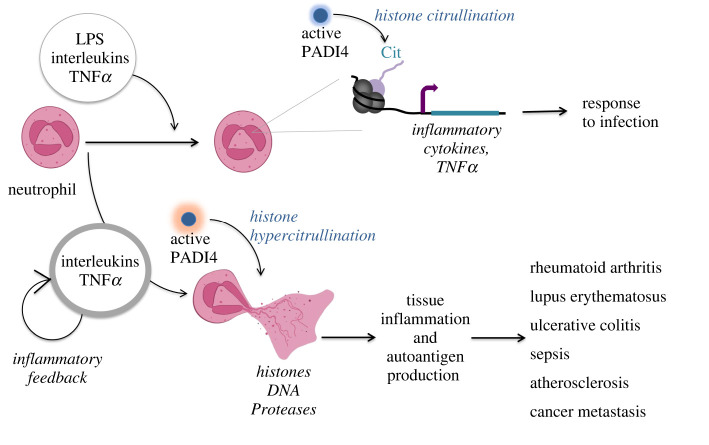
Modes and outcomes of PADI4 activation in the immune system. Schematic representation of the outcomes of PADI4 activation in neutrophils. Upon infection, active PADI4 can citrullinate histones to mediate transcriptional activation of inflammatory cytokines. Increased inflammatory signalling can lead to histone hypercitrullination and NETosis, which can cause tissue destruction and exacerbate a number of pathologies.

One of the best-established functions of protein citrullination is in immune responses and sterile inflammation (figure 2). PADI4 is most prominently expressed in neutrophils and other leucocytes and can mediate immune signalling via transcriptional and biochemical regulation of cytokines [74]. Furthermore, PADI4 is strongly activated during the innate immune response to infection and mediates NETosis, a process of inflammation-induced cell death, which involves profound decondensation and release of chromatin from neutrophils [119]. PADIs also impact adaptive immune responses through the regulation of T cell differentiation. As mentioned above, PADI2-mediated citrullination of transcription factors GATA3 and ROR*γ*t affects T cell responses by modulating the relative numbers of helper T cell sub-populations and, consequently, tissue inflammation [75]. Recent work suggested that differentiation of helper T cells is also affected by NET-associated extracellular histones [120], a process likely mediated by citrullination.

Citrullination is also inextricably associated with autoimmunity. The *Padi4* gene falls within a susceptibility locus for Rheumatoid Arthritis (RA), and certain single nucleotide polymorphisms are associated with the disease [14]. The adaptive immune system reacts specifically to citrullinated peptides of endogenous proteins and presentation of these modified peptides by Major Histocompatibility Complex (MHC) molecules on antigen-presenting cells elicit highly specific T cell responses [121], which are thought to be associated with autoimmunity. Citrullinated peptides have also been shown to bind with higher affinity to certain HLA alleles, which are associated with susceptibility to RA [122], providing a mechanistic basis for the strong links between citrullination and autoimmunity, however more recent work has demonstrated that this may not a universally applicable explanation [123]. Autoantibodies against citrullinated versions of such proteins (anti-citrullinated protein autoantibodies, or ACPA) serve as diagnostic and prognostic RA biomarkers and their presence is associated with faster progressing and more destructive disease [124], while similar autoantigens have also been associated with lupus erythematosus and other autoimmune disorders [67].

### 5.5. NETs and NET-associated pathologies

NETosis constitutes a fast and effective response to infection, however, the deregulation of this process and associated release of inflammatory cytokines, proteases and active PADIs to the extracellular space, are highly destructive for the surrounding tissue and have been associated with the exacerbation of a remarkable array of pathologies, including RA, lupus erythematosus, ulcerative colitis, inflammatory bowel disease, atherosclerosis, sepsis, type I diabetes and severe Covid-19 (reviewed recently in detail in [125]) (figure 2).

A large body of literature suggests that PADI4 activation is an essential step and operates in the early stages of NETosis [68,118,126,127]. However, detailed imaging methods developed recently have mapped the sequence of events that take place during NETosis and suggest that histone citrullination is a late, rather than initiating event [128,129]. More recently, it was suggested that citrullination does not mediate NETosis in neutrophils, but modulates the binding of histones to Toll-like receptors, thereby mediating downstream signalling [130]. Additionally, a significant number of studies have shown that NETosis can progress in a PADI4-independent manner [131,132]. While some of these studies may be complicated by the stimuli used to induce NETosis in the different studies [133,134], *in vivo* experiments that examined the role of PADI4 in NETosis associated with bacterial infection, diabetes, sepsis and cancer, among others, show that PADI4 inhibition interferes with the ability of neutrophils to release NETs [118,135,136]. As a result, PADI4 inhibition has been studied extensively as an approach to alleviate NET-associated pathologies that manifest in diabetes, sepsis, infection-induced lung injury, age-related cardiac fibrosis, deep-vein thrombosis and myocardial ischemia [137–142].

### 5.6. Cancer

A substantial body of work draws a strong association between PADI deregulation and cancer. Evidence suggests that PADIs can impact on tumour development through modulating cell signalling, transcription and the extracellular matrix (ECM), thereby regulating growth, apoptosis and the epithelial-to-mesenchymal transition. PADI2-mediated transcriptional regulation is associated with the development and metastasis of breast, gynecological and prostate cancers [60,143,144]. PADI4 is detected in the blood of cancer patients and in a large array of malignant tumours whose normal tissue and benign tumour counterparts lack PADI4 expression, while metastatic tumours exhibit significantly higher PADI4 expression than the corresponding primary tumours [49,90,145]. This suggests that cells that aberrantly upregulate or activate PADI4, either through genetic mutation or deregulation of PADI4-activating signalling pathways, have a growth advantage and are selected during cancer development and metastasis. This has been mechanistically attributed to PADI4-mediated remodelling of the ECM and initiation of metastatic colony formation [90]. In addition, PADI4-mediated regulation of gene transcription has been shown to interfere with apoptosis and growth arrest in cancer cell lines [146–148]. An expanding body of literature also suggests that PADI4 may have an indirect role in supporting cancer progression, through the promotion of NETs [149]. Importantly, these studies showed that loss of PADI4 in mice reduced both NETs formation and cancer metastasis [150,151]. The above studies suggest that when PADIs act to promote cancer their activation is co-opted by existing and emerging tumors rather than acting as a tumour-initiating event. However, some evidence also exists to suggest that PADI2 over-expression can act as a tumour initiating mechanism [152].

On the other hand, PADIs have also been suggested to have anti-tumour effects. PADI2 has been shown to mediate the action of an anti-cancer compound that acts through the dampening Wnt signalling [81], while PADI4 was shown to inhibit epithelial-to-mesenchymal transition [80]. Additionally, PADI4 has been suggested to act as a tumour suppressor as *Padi4-null* mice showed resistance to DNA damage-induced apoptosis in the thymus and H4R3 citrullination was associated with smaller tumour size in a cohort of non-small cell lung carcinoma patients [153]. However, this study did not detect a significant association between levels of H4R3 citrullination in the tumours and increase in patient survival. While PADIs cannot be broadly and unequivocally summarized as pro- or anti-tumourigenic factors, the field has amassed enough evidence to warrant the serious consideration of PADI inhibitors in cancer therapy, especially for late stage and metastatic cancers.

Beyond modulation of PADI activity as an approach to cancer therapy, an emerging body of work suggests that citrullination may be exploited in the development of anti-cancer vaccines [154]. Citrullinated epitopes of endogenous proteins such as vimentin have been found to be presented by cancer cells, in addition to antigen-presenting cells [155]. The authors demonstrated that immunization with a citrullinated vimentin peptide resulted in a specific T cell response and increased the survival of tumour bearing mice [156].

The studies discussed above collectively demonstrate that inhibition of PADIs, and PADI4 more specifically, holds significant promise as a therapy for a large array of clinical conditions. Indeed, significant effort has been devoted toward generating potent and specific PADI inhibitors [24,101,127]. The demonstration that loss of PADI4 function in the haematopoietic system, where it is most highly expressed, has no adverse effects for normal haematopoiesis in mice [46], suggests that PADI4 inhibition may be well tolerated and increases the urgency for the development of inhibitors suitable for clinical use.

## 6. Understanding regulation of PADI activity *in vivo*—how to exalt the virtues while keeping the vices at bay

Given the strong association between aberrantly high levels of citrullination and disease states such as autoimmunity, neurodegeneration and cancer [24], it is important to consider the mechanisms that govern PADI activation, how they may operate in different cellular contexts and how they may be deregulated in disease. An important open question in citrullination biology is how PADIs are regulated *in vivo*.

PADI1-4 are calcium-dependent enzymes and their activation mechanisms have been mapped in great detail *in vitro*, showing that calcium binding is an obligatory step in generating the active cleft [3]. PADI4 is bound by five calcium ions, which induce the activity of the enzyme by greater than 10^5^-fold [3]. Elegant structural and biochemical studies of PADI2 by Slade *et al.* have shown that calcium binding sites are bound by si calcium ions in a stepwise fashion and identified a calcium switch that exposes the enzyme active site and positions the catalytic cysteine, rendering the enzyme greater than 7 × 10^5^-fold more active [4]. The putative calcium switch residues are highly conserved among PADI1-4, suggesting that the calcium switch is a universal activation mechanism.

Within a cellular context, PADI activation is tightly regulated and it is possible for the enzyme to be expressed but catalytically inactive. For example, neutrophils express robust amounts of PADI4 but citrullination is undetectable until the cells are stimulated with a calcium ionophore or inflammatory stimuli such as TNF*α* and lipopolysaccharide (LPS) [157]. The calcium concentration necessary to activate PADIs *in vitro* is at least 10-fold higher than reported intracellular calcium concentrations, which reach up to 10 µM [158]. While activation is likely to be temporally and spatially controlled, it is as yet unclear whether PADIs are always activated as a result of local calcium influx or whether their activation is subject to allosteric regulation. Opening of calcium channels can lead to local calcium concentrations in the micromolar range [159,160] and this would be sufficient to activate catalysis. Indeed, it is known that some of the inflammatory stimuli that lead to PADI4 activation in neutrophils, such as chemotactic peptides, incite a calcium influx and a similar mechanism has been attributed to progesterone-dependent PADI2 activation in breast cancer cells [114,161]. However, it is unclear how PADIs may be recruited to the proximity of high calcium areas, especially when considering PADI activation within the nucleus, as in the case of PADI4- or PADI2-mediated citrullination of histone proteins [54,55].

An alternative mechanism involves allosteric regulation. Binding to a cofactor, such as an interacting protein or complex, may elicit a conformational change that mimics the calcium switch by exposing the catalytic cysteine, thus rendering catalysis possible in intracellular calcium concentrations. Support for this mechanistic scenario comes from studies that showed that RA-specific autoantibodies against PADI4 bind near the calcium switch site and lower the calcium concentration required for activation [162,163]. Similarly, it is plausible that the cell signalling pathways that are engaged under conditions of PADI activation result in PTMs that confer a similar structural change. It has been suggested that autocitrullination of PADI4 occurs during neutrophil activation and alters its catalytic activity [164], while the heavy involvement of kinases within the inflammatory signalling pathways that are known to lead to PADI activation makes it likely that phosphorylation events and other PTMs have a regulatory role. In breast cancer cells, PADI4-mediated citrullination of the transcription factor Elk-1 happens upon stimulation with Epidermal Growth Factor (EGF), suggesting that PADI4 may be activated by a component of EGF signalling [16], although it is yet unclear from this study whether it is the catalytic activity of PADI4, or its association with Elk-1 that is regulated in this manner. Similarly, ATP-dependent PADI2 activation in mast cells is mediated by the kinases p38 MAPK and PKC, supporting the possibility that PADI2 activation is modulated by phosphorylation [165].

Understanding the biochemical mechanisms that lead to PADI activation requires the identification of the precise conditions and cell stimuli that elicit a citrullination response. In this respect, the fields of neutrophil activation and inflammatory signalling offer fertile ground for investigation, although significant progress in this regard has been impeded by the seeming heterogeneity and differing outcomes of neutrophil responses depending on the experimental context, as well as variability in the antibody reagents typically used to access PADI4 activation [166].

A recently reported context of PADI4 activation is likely to provide additional research avenues in this area. Wang *et al.* discovered that PADI4 is activated under hypoxic conditions and mediates hypoxia-inducible factor (HIF) dependent transcriptional regulation in cancer cells [167]. Although the activation is closely associated with hypoxia-mediated upregulation of PADI4, kinetic experiments in this study also report conditions under which PADI4 activation is observed within a few hours of the hypoxic stimulus and before any appreciable upregulation of the protein. This suggests that hypoxia leads to the enzymatic activation of PADI4, as well as its upregulation. Disentangling the catalytic and transcriptional/translational regulation of PADIs will be a key step toward understanding mechanisms of allosteric regulation and hypoxic stimuli may provide a cleaner system than growth promoting or inflammatory signalling.

A study published earlier this year identified that cytomegalovirus infection elicits upregulation and activation of PADI2 and PADI4 and consequent citrullination of a wide range proteins [168]. The timing of PADI activation in this context makes it difficult to ascertain whether viral infection regulates the catalytic activity of PADIs, or whether the citrullination observed is due to increased levels of the enzymes. However, more detailed temporal studies in this context may provide an additional useful system in which to study PADI activation.

An alternative, but not mutually exclusive, possibility is that PADIs are subject to inhibitory PTMs, which are removed or reversed under activating conditions. A study by Chang *et al.* reported that association with the protein tyrosine phosphatase PTPN22 suppresses the catalytic activity of PADI4 and subsequent NETosis [169]. Although in this context PADI4 inhibition is not mediated via the phosphatase activity of PTPN22, it is possible that association with other phosphatases or other PTM-reversing regulates PADI activity.

An exciting possibility is that detailed understanding of the mechanistic principles of PADI activation and how they differ between physiological and disease conditions may enable the design of a next generation of therapeutics based on modulators that will achieve precise perturbation or activation of citrullination. It will therefore be a valuable endeavour for the field to pursue studies that will delineate the cellular conditions, signalling pathways and biochemical mechanisms that underlie PADI regulation in physiology and disease.

## 7. Discussion

PADI enzymes are ubiquitous across vertebrates and are becoming increasingly appreciated as key regulators in many aspects of mammalian physiology. They are absent from classical model organisms such as yeast, worms and flies [25] and, arguably, this has impeded progress into understanding the physiological functions of citrullination, as the field wasn't afforded the rich information that can be derived from systematic genetic screens. The discovery that PADIs arose in animals by horizontal gene transfer leads us to think about citrullination and its roles in animal biology in a new light. The fact that the PADI sequence was not only retained throughout vertebrate evolution, but duplicated multiple times, suggests that citrullination confers a fitness or survival advantage to animals. While there is still a lot to understand regarding the physiological functions of PADIs, an explanation for their retention may be the role of citrullination in the defense against infection. Indeed, it has been suggested that genes that are horizontally transferred from bacteria may augment the innate immune functions of eukaryotes [170]. Alternatively, the emerging role of citrullination in mammalian embryonic development [10,109,110] may explain the selective pressure for retaining the sequence. A notable insight into this question is offered by a recent study in fish. Golenberg *et al*. deleted the single PADI in zebrafish and found that this protein regulates wound healing and fin regeneration [171]. It is not yet known how the PADI-null zebrafish respond to infection and it is possible that PADIs have acquired different roles, even throughout animal evolution. However, we can safely assume, and indeed we are starting to appreciate, that some of their functions are of fundamental importance in animal physiology.

The future is undoubtedly bright for citrullination research and we can predict that the field will see yet greater progress in the short- and mid-term, as researchers are building ever more specific and powerful tools for the manipulation of PADIs and the detection of their activation, as well as animal models where their function is perturbed. It has been pleasing to see that citrullination was highlighted as one of the PTMs that are likely to have a significant impact in the field of signal transduction, in an editorial on ‘The Future of Signaling’ a few years back [172].

## 8. Outlook

Some questions are largely unresolved and it is the author's view that the following are of immediate importance: Firstly, is citrullination a *bona fide* signal transduction mechanism, one that mediates the translation of environmental cues into transcriptional and epigenetic changes in normal physiology? Secondly, what determines ‘normal’ versus ‘pathogenic’ levels of citrullination in a tissue? Is it the magnitude, or the nature of the activating signal that is altered in the various pathologies exacerbated by aberrant citrullination? If it is the mechanism, can we exploit this in disease therapy? Thirdly, the long-standing question on the elusive reversing mechanism: after a stimulus that induces citrullination, for example of histones, the citrulline mark disappears with time [54]. Is this due to the existence of a citrullination ‘eraser’, akin to a phosphatase or deacetylase? Or is citrullination only lost through degradation and recycling of the modified protein? And lastly, can we map specific residues or regions within PADIs that may be perturbed to achieve certain outcomes, for example a constitutively active PADI, or one that only responds to certain stimuli? We can dare to speculate that, by continuing to build upon current knowledge and moving towards precise biochemical understanding of PADI activation and substrate engagement mechanisms, it will be possible to answer these outstanding questions.

## Data Availability

This article has no additional data.
